# Intradialytic Parenteral Nutrition in Patients on Hemodialysis: A Multicenter Retrospective Study

**DOI:** 10.3390/nu16234018

**Published:** 2024-11-24

**Authors:** Marta Arias-Guillén, Juan Carlos González, Loreley Betancourt, Elisabeth Coll, Silvia Collado, Bárbara Romano-Andrioni, Ascensión Lupiañez-Barbero, Julia Garro, Verónica Duarte, Jordi Soler-Majoral, Jordi Calabia

**Affiliations:** 1 Renal Transplantation and Nephrology Department, Hospital Clinic Barcelona, 08036 Barcelona, Spain; 2 Nephrology Department, Hospital de Mollet, 08100 Barcelona, Spain; jc.gonzalez@fsm.cat; 3 Nephrology Department, Corporació Sanitaria Parc Taulí, 08208 Barcelona, Spain; lorelaybc@yahoo.es; 4 Nephrology Department, Fundació Puigvert, 08025 Barcelona, Spain; elisabetcopi@gmail.com; 5 Nephrology Department, Hospital del Mar-Parc de Salut Mar, 08003 Barcelona, Spain; scollado@parcdesalutmar.cat; 6 Nutrition and Dietetic Unit, Hospital Clinic Barcelona, 08036 Barcelona, Spain; 7 Diaverum Renal Services, Nutrition, Catalonia, 08024 Barcelona, Spain; ascension.lupianez@diaverum.com; 8 Nephrology Department, Hospital Universitari Joan XXIII, 43005 Tarragona, Spain; jgarro.hj23.ics@gencat.cat; 9 Nephrology Department, Hospital de Terrassa, 08227 Terrassa, Spain; veronica.duarte.gallego@gmail.com; 10 Nephrology Department, Hospital Germans Trias I Pujol, 08916 Barcelona, Spain; jsoler.germanstrias@gencat.cat; 11 Nephrology Department, Hospital Universitari Dr. J Trueta, 17007 Girona, Spain; jcalabia.girona.ics@gencat.cat

**Keywords:** intradialytic parenteral nutrition, protein-energy wasting, hemodialysis, chronic kidney disease, malnutrition inflammation score

## Abstract

Background and Objective: To evaluate the effectiveness and safety of intradialytic parenteral nutrition (IDPN) on different nutritional outcomes. Methods: This was a retrospective analysis for a “routinely collected data bank” in a multicenter cohort, conducted on consecutive malnourished or at-risk of malnutrition patients with chronic kidney disease on hemodialysis who underwent IDPN with a three-in-one parenteral nutrition formula for a period ≥ 2 weeks. The primary endpoint was the mean change in the malnutrition inflammation score (MIS) score between baseline and the last follow-up visit on IDPN. Results: Fifty-six patients were included. The mean age was 72.4 ± 12.0 years, and 24 (42.9%) were women. In the overall study sample, MIS significantly decreased from 16.4 (95%CI: 15.3–17.65) at baseline to 14.3 (95%CI: 12.8–15.8) at the last follow-up visit on IDPN (*p* = 0.0019). Fifteen (26.8%) patients achieved a MIS reduction ≥ 5 points after IDPN. As compared to baseline, IDPN significantly reduced the proportion of patients with protein-energy wasting (PEW) (89.3% versus 66.1%, respectively, *p* = 0.0023). Regarding analytical parameters, serum albumin (*p* = 0.0003) and total proteins (*p* = 0.0024) significantly increased after IDPN administration. Throughout the study’s follow-up period, 45 (80.4%) patients reported experiencing some type of adverse event. Conclusions: IDPN was associated with a significant improvement in the nutritional profile. Notably, our research found that the administration of IDPN over a duration > 3 months significantly improved the nutritional status of patients evaluated by the MIS test.

## 1. Introduction

Patients with chronic kidney disease (CKD), particularly those on hemodialysis (HD), are prone to malnutrition [1,2]. While multiple factors contribute to the onset of malnutrition, they can broadly be classified as either iatrogenic or non-iatrogenic in nature. Iatrogenic factors arise as unintended consequences of dialysis in end-stage CKD patients, whereas non-iatrogenic factors emerge independently, driven by conditions associated with the progression of CKD but unrelated to the primary therapeutic intervention [1,2]. This nutritional status impairment has been associated with worse clinical outcomes, greater morbidity and mortality, and lower quality of life [3,4]. Moreover, there is a progressive decline in nutritional status of CKD patients, which leads to depletion of protein-energy stores. This condition was termed “protein-energy wasting” (PEW), which has a high prevalence among CKD patients on HD and is often associated with reduced functional capacity [2,5,6].

When dietary counseling strategy does not attain the recommended energy and protein requirements, a comprehensive combination of nutritional strategies is needed to provide adequate amounts of protein and energy. This comprehensive approach, which includes oral nutritional supplements (ONS) and intradialytic parenteral nutrition (IDPN), should be considered as a viable option to improve nutritional status [7,8].

According to the ESPEN and KDOQI guidelines, IDPN is indicated in non-critically ill patients with CKD on HD who are malnourished or at risk of malnutrition and cannot meet their nutritional needs with oral diet alone [8,9].

Some studies have reported conflicting results about the advantages of IDPN, mainly due to un-clear selection criteria of participants, lack of concurrent comparators, and the heterogeneity of IDPN admixtures [10,11,12]. Nevertheless, current evidence suggests that IDPN significantly improved the nutritional status of CKD patients on HD, as well as spontaneous oral intake [13,14,15].

The evidence evaluating the efficacy and safety of IDPN in daily clinical practice is limited. We have previously demonstrated a high prevalence of PEW in our area using different nutritional scores, with the malnutrition inflammation score (MIS) being the one with more sensitivity to diagnose PEW [16]. For this reason, we wanted to analyze the reality of our Autonomous Community (Catalonia).

This study aimed to evaluate the effect of IDPN on different nutritional outcomes, including the MIS, biochemical parameters, and body composition data. In addition, we also assessed the safety of IDPN in terms of incidence and frequency of adverse events (AEs).

## 2. Materials and Methods

### 2.1. Study Design

This was a retrospective analysis for a “routinely collected data bank” in a multicenter cohort, conducted on consecutive malnourished or at-risk of malnutrition patients with CKD on HD who underwent IDPN for a period ≥ 2 weeks in one of the eight Catalan hospitals participating in this study.

The study protocol was approved by the Ethic Committee of the Hospital of Girona as a new version of the Nutrendial project (Protocol number: 2016.141, Approval Date: 27 September 2016) [16].

This study complied with the Good Clinical Practice/International Council for Harmonization Guidelines, the Declaration of Helsinki, and all applicable country-specific regulations governing the conduct of clinical research, depending on which provided greater protection to the individual.

Written informed consents were obtained from all the patients involved in this study. Any information that could lead to an individual being identified has been encrypted or removed as appropriate to guarantee their anonymity.

### 2.2. Study Participants

This study included patients, aged ≥ 18 years; with a CKD stage 5 [9] and ≥90 days on HD; with malnutrition or at risk of malnutrition; MIS ≥ 8; ability to give written informed consent; availability, willingness, and sufficient cognitive awareness to comply with the procedures, indications of the investigator, and schedule of the exam.

Patients with cancer or other severe disease who had a life expectancy < 12 months; clinically significant infection; pregnant (or not taking contraceptive measures) or nursing women; or those who received treatment with IDPN within 3 months prior to the baseline visit were excluded from this study.

### 2.3. IDPN Composition

Patients received a 3-in-1 parenteral nutrition formula (OlimelN9^®^, Baxter Healthcare Corporation Deerfield, IL 60015 USA) consisting of glucose, essential and non-essential amino acids, and lipid emulsion.

The IDPN formula must meet the following conditions: proteins (0.8–1.2 g/kg weight); non-protein energy (1000–1200 Kcal); carbohydrates (150–175 g); lipids (40–50 g); vitamins; and trace elements.

IDPN was infused at a constant rate during 4 h but not exceeding 250 mL/h via a venous drip chamber of the HD machine using an infusion pump.

### 2.4. Study Groups

To compare the effect of time on IDPN on clinical and nutritional outcomes, patients were stratified into two groups: patients with ≤3 months and patients with >3 months on IDPN [14].

### 2.5. Study Outcomes

The primary endpoint was the mean change in the MIS between baseline and the last follow-up visit on IDPN.

The MIS comprises ten components, each representing distinct facets of the malnutrition–inflammation complex, including weight change, dietary intake, gastrointestinal symptoms, functional capacity, comorbidities, fat stores, muscle wasting, body mass index, serum albumin concentration, and total iron-binding capacity. Each component is graded on a severity scale of four levels, ranging from 0 (normal) to 3 (severely abnormal). The aggregate score from all ten MIS components can vary from 0 (normal) to 30 (severely malnourished), with higher scores indicating a more severe degree of malnutrition and inflammation [16].

The secondary end-points were the mean changes in biochemical parameters (albumin, prealbumin, creatinine, cholesterol, transferrin, and C-reactive protein); body composition analysis (weight and body mass index); and subjective global assessment (SGA score). SGA assesses five elements of medical history—weight change, dietary intake, gastrointestinal symptoms, functional capacity, and the disease’s impact on nutritional needs—along with three components of a concise physical examination, which includes indicators of fat and muscle wasting and nutrition-related alterations in fluid balance, to assess an individual’s nutritional status [17].

Normalized protein catabolic rate (nProtCatRate) was calculated according to the formula proposed by Depner and Daugirdas [18].

Additionally, the difference in the prevalence of PEW between baseline and the last follow-up visit was evaluated. Finally, the incidence of any adverse event was recorded and evaluated.

### 2.6. Statistical Analysis

Statistical analysis was performed with the MedCalc^®^ Statistical Software version 22.021 (MedCalc Software Ltd., Ostend, Belgium; https://www.medcalc.org; 2024. Last accessed 17 October 2024).

Prior to this study, it was determined that at least 54 patients were required to detect a difference of 2 units in the mean MIS, at a significance level of 0.05, with a power of 90% and assuming a standard deviation of 4.5 units.

We conducted two different efficacy analyses. The first one included all patients who received IDPN for a period ≥ 2 weeks, while the second analysis included patients who received IDPN for a period ≥ 3 months.

The Shapiro–Wilk test was used for assessing quantitative variables’ normality.

In normally distributed variables, the two-tailed Student’s *t*-test was used to analyze the changes in continuous variables, while if such variables were not normally distributed, the Wilcoxon test was used.

The McNemar test was used to assess the PEW rate difference between baseline and last follow-up visit.

Categorical variables were compared using a chi-square test and Fisher’s exact test, as needed. A *p*-value of less than 0.05 was considered significant.

## 3. Results

**Figure 1:**
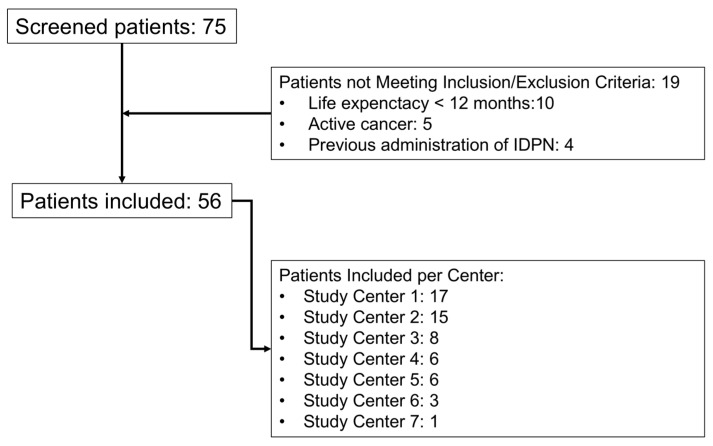
Flow chart of the patients included in this study. IDPN: Intradialitic parenteral nutrition. Center 1: Hospital Clinic; Center 2: Hospital de Mollet; Center 3: Fundació Puigvert; Center 4: Hospital del Mar-Parc de Salut Mar; Center 5: Corporació Sanitaria Parc Taulí; Center 6: Hospital Universitari Dr. J Trueta; and Center 7: Hospital Universitari Joan XXIII.

A total of 75 patients were initially evaluated, of which 19 did not meet the inclusion/exclusion criteria (10 due to life expectancy < 12 months, 5 due to active cancer, and 4 due to previous IDPN administration). Fifty-six patients met all the inclusion criteria and none of the exclusion criteria and were included in the analysis (see Figure 1).

### 3.1. Baseline Demographic, Clinical, Analytical, and Nutritional Characteristics

The mean age of the study sample was 72.4 ± 12.0 years, and 24 (42.9%) were women. The mean MIS was 16.4 ± 4.2, and, according to the SGA, 37 (67.3%) patients had experienced weight loss > 10% (SGA grade C). At baseline, 50 (89.3%) had a PEW. The main demographic, clinical, analytical, and nutritional characteristics of the study sample are shown in Table 1.

### 3.2. Malnutrition Inflammation Score

**Figure 2:**
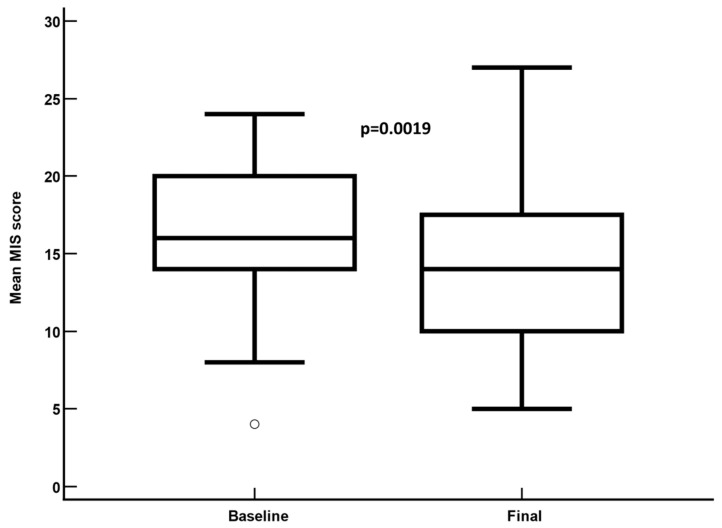
Mean malnutrition inflammation score at baseline and at the last follow-up visit on intradialytic parenteral nutrition. Mean difference: −2.1 ± 4.8; 95% confidence interval: −3.5 to −8.0; *p* = 0.0019.

In the overall study sample, the mean (95%CI) MIS significantly decreased from 16.4 (from 15.3 to 17.65) at baseline to 14.3 (from 12.8 to 15.8) at the last follow-up visit on IDPN (mean difference: −2.1; 95%CI: from −3.4 to −0.8; *p* = 0.0019) (Table 2, Figure 2). Fifteen (26.8%) patients achieved a MIS reduction ≥ 5 points after IDPN.

This finding was further validated in the analysis that included patients on IDPN ≥ 3 months (*n* = 32), revealing a statistically significant decrease in MIS (mean difference: −2.2; 95%CI: from −3.6 to −0.73; *p* = 0.0045) as shown in Table 3.

### 3.3. Other Analytic- and Nutrition-Related Variables

Regarding analytical parameters, serum albumin (mean difference: 0.4 g/dL; 95%CI: from 0.16 to 0.52 g/dL; *p* = 0.0003) and total proteins (mean difference: 3.3 g/L; 95%CI: from 1.2 to 5.4 g/L; *p* = 0.0024) significantly increased after IDPN administration (Table 2). These results were also confirmed in those patients on IDPN ≥ 3 months by the per protocol analysis (Table 3).

The proportion of patients with PEW at baseline (89.3%; 50/56) was significantly reduced after administration of IDPN (66.1%; 37/56) (mean difference: −23.2%; 95%CI: from −36.3% to 10.1%; *p* = 0.0023, McNemar test).

Regarding SGA, at baseline, the proportion of patients categorized as A, B, or C were 1.8%, 30.9%, and 67.3%, respectively, while after administration of intradialytic parenteral nutrition there was a significant improvement to 3.6%, 43.6%, and 52.7%, respectively; *p* = 0.0064 (chi-square test).

The BMI did not significantly change after IDPN administration.

### 3.4. Nutritional and Analytical Parameters Depending on IDPN Duration

To investigate the impact of IDPN duration on both nutritional and analytical outcomes, the study participants were divided into two categories: individuals who underwent IDPN for less than 3 months (median time: 2.4 months, 95%CI: from 2.0 to 2.6 months), and those who underwent IDPN ≥ 3 months (median time: 6.6 months, 95%CI: from 4.4 to 7.1 months) (see Table 4).

Among patients who underwent IDPN for less than 3 months, significant improvements were observed in albumin and total protein levels, although no change was noted in the MIS. Conversely, individuals who underwent IDPN ≥ 3 months exhibit significant enhancements in their MIS. Furthermore, this cohort demonstrated noteworthy improvements in albumin, prealbumin, C-reactive protein, and total protein levels.

However, there were no significant differences between groups in both nutritional and analytic variables (Table 4).

### 3.5. Safety

Throughout the study’s follow-up period, 45 (80.4%) patients reported experiencing some type of adverse event, none of them in relation to the administration of IDPN (Table 5).

Throughout the study’s follow-up period, 30 (53.6%) patients had infection, and 36 (64.3%) patients required hospitalization. Nevertheless, it is important to note that none of these incidents were associated with the administration of IDPN.

## 4. Discussion

The current study evaluated the impact of the IDPN on the nutritional status of patients with CKD on HD. The results of this study found a significant improvement in the MIS as well as in the albumin and total protein levels after IDPN administration. In addition, the proportion of patients with PEW was significantly reduced after IDPN. Regarding safety, 36 patients were admitted to the hospital, although no hospital admissions were reported in relation to the administration of IDPN.

Assessing the response to therapy is crucial in the patient’s care, and different parameters have been suggested to evaluate the efficacy of IDPN over time, including pre-dialysis albumin and prealbumin levels, SGA, body stores assessment by bioelectrical impedance, or handgrip strength [14]. In addition, the malnutrition inflammation score (MIS) and subjective global assessment (SGA) have been proposed as valuable tools for assessment of nutritional status in patients on HD, especially in clinical settings where wasting characteristics need to be considered [19]. Moreover, MIS was strongly associated with nutrition indicators, with a higher MIS indicating an increased risk of unfavorable clinical outcomes in patients with CKD undergoing dialysis and kidney transplantation [3,20,21,22,23,24]. Therefore, it could be hypothesized that a significant reduction in the MIS would be associated with better clinical outcomes, although the design of this study does not allow for this claim.

As per the updated KDOQI guideline of 2020, serum albumin levels have been recognized as the most reliable predictor of mortality among biochemical indicators used to assess PEW in HD patients [9]. The results of an epidemiological study indicated that a rise in serum albumin of 0.2–0.3 g/dL correlated with a 20% reduction in the likelihood of death, even after adjusting for different covariates [25].

As compared to baseline values, we observed a statistically significant improvement in the albumin levels after administering IDPN, with a mean increment of 0.4 ± 0.7 g/dL. This finding agreed with those reported by different randomized controlled trials, which found that IDPN significantly increased serum albumin [13,26,27,28,29].

Prealbumin is often regarded as a more sensitive marker due to its shorter half-life compared to albumin, which makes it more sensitive to fluctuations in protein-energy status compared to albumin, as its concentration closely mirrors recent dietary intake rather than providing a comprehensive evaluation of overall nutritional status [30].

Finally, our study observed a significant decrease in the proportion of patients with PEW after the administration of IDPN, indicating the clear beneficial impact of the therapy on the nutritional status of the patients.

Although this study suggested that administering IDPN for less than three months yielded a significant improvement in albumin and total protein values, the administration of IDPN for a period ≥ 3 months resulted in superior analytical and nutritional outcomes. In addition, C-reactive protein decreased significantly only in the group of patients who received IDPN for a period ≥ 3 months. This observation suggests that administering IDPN for a period ≥ 3 months may exert anti-inflammatory effects.

While the ideal duration of IDPN may vary based on individual patient considerations, it is reasonable to propose that IDPN therapy during HD should persist for a minimum of three months to facilitate substantial evaluation [14]. After this timeframe, it is advisable to reassess the nutritional status to determine if IDPN is still needed.

In terms of safety, 45 patients (80.4%) reported experiencing some form of adverse event, with hospital admissions and infections being the most significant among them. In this sense, it should be considered that patients included in this study have presented, previously or at the time of inclusion, a clinical situation that favors malnutrition, such as infectious illness, cardiovascular event, surgical procedure, or prolonged hospitalization, among others. For this reason, the adverse events collected during the follow-up cannot be related to IDPN. IDPN has not been associated with a significant increase in infection rates, unlike other parenteral nutrition methods that require a separate administration route, as IDPN utilizes the dialysis circuit, minimizing the risk of infection [31].

The current study has several limitations that should be considered when interpreting its results. Despite appropriate statistical calculations, it is important to note that the number of patients in this study remained relatively low. On the other hand, this is an observational study, and for ethical reasons there was no control group. Furthermore, the findings are confined to a specific geographic region, potentially limiting the generalizability of the results.

## 5. Conclusions

The results of this real-life study revealed that the administration of IDPN was associated with a significant improvement in the nutritional status of patients with CKD on HD.

IDPN showed an improvement in MIS, serum albumin, and total protein levels, as well as a significant reduction in the proportion of patients with PEW. Notably, our research found that the administration of IDPN over a duration > 3 months significantly improved the nutritional status of patients.

Further research with longer follow-up periods and larger patient cohorts is crucially needed to confirm the beneficial role of IDPN supplementation in enhancing clinical outcomes among the HD population with PEW.

## Figures and Tables

**Table 1:** Baseline demographic, clinical, analytical, and nutritional characteristics of the study sample.

Variable	N = 56
Age, yrs	
Mean ± SD	72.4 ± 12.0
Median (IqR)	75.0 (65.5 to 81.0)
Sex, n (%)	
Women	24 (42.9)
Men	32 (57.1)
DM, n (%)	
No	30 (53.6)
Yes	26 (46.4)
Charlson index	
Mean ± SD	8.8 ± 2.8
Median (IqR)	8.0 (7.0 to 10.0)
BMI, kg/m^2^	
Mean ± SD	21.6 ± 4.7
Median (IqR)	21.1 (17.7 to 24.5)
MIS	
Mean ± SD	16.4 ± 4.2
Median (IqR)	16.0 (14.0 to 20.0)
PEW, n (%)	
No	6 (10.7)
Yes	50 (89.3)
SGA, n (%) *	
Weight loss < 5%	1 (1.8)
Weight loss 5–10%	17 (30.9)
Weight loss > 10%	37 (67.3)
Albumin, g/dL	
Mean ± SD	3.04 ± 0.60
Median (IqR)	3.05 (2.65 to 3.30)
Prealbumin, g/L	
Mean ± SD	0.18 ± 0.09
Median (IqR)	0.15 (0.12 to 0.21)
Creatinine, mg/dL	
Mean ± SD	4.9 ± 1.9
Median (IqR)	4.5 (3.6 to 6.2)
Cholesterol, mg/dL	
Mean ± SD	128.5 ± 46.1
Median (IqR)	120.0 (97.8 to 154.3)
Transferrin, mg/dL	
Mean ± SD	139.6 ± 66.2
Median (IqR)	121.0 (100.0 to 150.0)
CRP, mg/dL	
Mean ± SD	12.5 ± 37.5
Median (IqR)	3.9 (1.2 to 8.0)
Total Protein, g/L	
Mean ± SD	51.9 ± 18.2
Median (IqR)	56.0 (49.0 to 63.0)
nProtCatRate, g/kg/day **	
Mean ± SD	1.00 ± 0.39
Median (IqR)	1.07 (0.76 to 1.26)

* Fifty-five patients. ** Seventeen patients. yrs: years; SD: standard deviation; IqR: interquartile range; DM: diabetes mellitus; BMI: body mass index; MIS: malnutrition inflammation score; PEW: protein-energy wasting; SGA: subjective global assessment; CRP: C-reactive protein; nProtCatRate: normalized protein catabolic rate.

**Table 2:** Mean change in quantitative nutritional and analytical parameters from baseline to the end of intradialytic parenteral nutrition (IDPN) in the overall study sample. Statistical significance was calculated using the two-way paired Student’s *t*-test.

Variable (*n* = 56)	Baseline	Final	Mean Difference (95%CI)	*p*
	Mean (95%CI)	Mean (95%CI)		
MIS	16.4 (15.3 to 17.65)	14.3 (12.8 to 15.8)	−2.1 (−3.4 to −0.8)	0.0019
BMI, kg/m^2^	21.6 (20.3 to 22.8)	21.8 (20.6 to 22.9)	0.25 (−0.15 to 0.64)	0.2167
Albumin, g/dL	3.0 (2.9 to 3.2)	3.4 (3.2 to 3.5)	0.4 (0.16 to 0.52)	0.0003
Prealbumin, g/L	0.18 (0.14 to 0.19)	0.43 (0.19 to 0.87)	0.25 (−0.17 to 0.70)	0.2208
Creatinine, mg/dL	4.9 (4.4 to 5.4)	5.1 (4.6 to 5.6)	0.22 (−0.34 to 0.78)	0.4332
Cholesterol, mg/dL ^a^	130.2 (117.4 to 142.9)	146.4 (134.2 to 158.7)	16.3 (2.2 to 30.4)	0.0242
Transferrin, mg/dL ^b^	137.5 (119.5 to 155.5)	138.4 (124.2 to 152.7)	0.96 (−14.6 to 16.5)	0.9016
CRP, mg/dL	12.5 (2.5 to 23.0)	5.0 (2.8 to 7.1)	−7.5 (−16.5 to 0.94)	0.0792
Total Protein, g/L ^a^	51.2 (46.2 to 56.2)	54.1 (48.9 to 59.2)	2.9 (0.5 to 5.4)	0.0215
nProtCatRate, g/kg/d ^c^	1.0 (0.8 to 1.2)	1.2 (0.9 to 1.4)	0.2 (−0.1 to 0.4)	0.1375

^a^ Three missing values. ^b^ Five missing values. ^c^ Data from 17 patients. CI: confidence interval; MIS: malnutrition inflammation score; BMI: body mass index; CRP: C-reactive protein; nProtCatRate: normalized protein catabolic rate.

**Table 3:** Mean change in quantitative nutritional and analytical parameters from baseline to the end of intradialytic parenteral nutrition (IDPN) in patients on IDPN ≥ 3 months. Statistical significance was calculated using the two-way paired Student’s *t*-test.

Variables (*n* = 32)	Baseline	Final	Mean Difference (95%CI)	*p*
	Mean (95%CI)	Mean (95%CI)		
MIS	17.1 (15.7 to 18.6)	14.9 (13.1 to 16.8)	−2.2 (−3.6 to −0.73)	0.0045
BMI, kg/m^2^	20.6 (19.1 to 22.1)	21.0 (19.6 to 22.4)	0.44 (−0.11 to 0.99)	0.1129
Albumin, g/dL	3.1 (2.8 to 3.3)	3.5 (3.3 to 3.9)	0.41 (0.13 to 0.68)	0.0044
Prealbumin, g/L	0.15 (0.13 to 0.17)	0.20 (0.16 to 0.24)	0.05 (0.01 to 0.10)	0.0246
Creatinine, mg/dL	4.8 (4.1 to 5.5)	5.4 (4.6 to 6.2)	0.59 (−0.19 to 1.36)	0.1326
Cholesterol, mg/dL ^a^	126.3 (109.4 to 143.9)	147.1 (130.4 to 163.9)	20.5 (−0.4 to 41.4)	0.0542
Transferrin, mg/dL ^b^	137.7 (122.4 to 152.9)	139.5 (112.0 to 167.1)	1.8 (−24.8 to 28.5)	0.8874
CRP, mg/dL	8.8 (3.9 to 13.6)	4.2 (2.0 to 6.4)	−4.6 (−8.3 to −0.85)	0.0178
Total Protein, g/L ^a^	54.5 (49.7 to 59.3)	58.7 (53.9 to 63.5)	4.2 (0.6 to 7.8)	0.0227
nProtCatRate, g/kg/d ^c,^*	1.20 (0.95 to 1.46)	1.28 (0.88 to 1.68)	0.08 (−0.24 to 0.40)	0.7794 *

^a^ Two missing values. ^b^ Four missing values. ^c^ Data from nine patients. * Wilcoxon test. CI: confidence interval; MIS: malnutrition inflammation score; BMI: body mass index; CRP: C-reactive protein; nProtCatRate: normalized protein catabolic rate.

**Table 4:** Overview of the mean change in quantitative nutritional and analytical parameters from baseline to the end of intradialytic parenteral nutrition (IDPN) for patients with ≤3 months and patients with >3 months on IDPN. Statistical significance was determined using the Mann–Whitney test.

Variable	IDPN < 3 Months (*n* = 24)		IDPN ≥ 3 Months (*n* = 32)		Mean Difference Between Groups (95%CI)	*p* ^b^
	Mean (95%CI) Difference from Baseline	*p* ^a^	Mean (95%CI) Difference from Baseline	*p* ^a^		
MIS	−1.9 (−4.3 to 0.48)	0.1032	−2.2 (−3.6 to −0.7)	0.0061	0.27 (−2.33 to 2.87)	0.7210
BMI, kg/m^2^	0.01 (−0.58 to 0.59)	0.6022	0.44 (−0.11 to 1.00)	0.0716	−0.44 (−1.23 to 0.35)	0.1622
Albumin, g/dL	0.25 (0.03 to 0.48)	0.0329	0.41 (0.14 to 0.68)	0.0058	0.20 (−0.15 to 0.56)	0.4144
Prealbumin, g/L	0.04 (−0.02 to 0.10)	0.0969	0.05 (0.01 to 0.10)	0.0206	0.01 (−0.01 to 0.09)	0.1401
Creatinine, mg/dL	−0.25 (−1.08 to 0.58)	0.9430	0.59 (−0.19 to 1.36)	0.1671	0.84 (−0.28 to 1.96)	0.3462
Cholesterol, mg/dL	10.8 (−8.4 to 30.0)	0.2294	20.5 (−0.40 to 41.4)	0.1413	9.7 (−18.8 to 32.3)	0.9356
Transferrin, mg/dL	4.4 (−9.9 to 18.7)	0.5310	1.8 (−24.8 to 28.5)	0.8874	−2.6 (−27.7 to 22.2)	0.6917
CRP, mg/dL	−1.6 (−5.8 to 2.6)	0.3391	−4.6 (−8.3 to −0.9)	0.0148	−3.2 (−9.1 to 2.7)	0.3270
Total Protein, g/L	1.2 (0.2 to 4.6)	0.0354	4.2 (0.6 to 7.8)	0.0227	3.0 (−1.9 to 7.9)	0.2277
nProtCatRate, g/kg/d	0.27 (−0.14 to 0.69)	0.1077	0.08 (−0.24 to 0.40)	0.7794	−0.19 (−0.66 to 0.28)	0.2682

^a^ Wilcoxon test. ^b^ Mann–Whitney test. IDPN: intradialytic parenteral nutrition; CI: confidence interval; MIS: malnutrition inflammation score; BMI: body mass index; CRP: C-reactive protein; nProtCatRate: normalized protein catabolic rate.

**Table 5:** Incidence of adverse events throughout the study follow-up.

Adverse Event *	N (%)
Overall	45 (80.4)
Infections	30 (53.6)
Hospitalization	36 (64.3)
Number of hospital admissions	
1	27 (48.2%)
2	8 (14.3)
3	0 (0.0)
4	1 (1.8)
Falls	14 (25.0)
Number of falls	
1	11 (19.6)
2	3 (5.4)

* Patients may have reported more than one adverse event throughout this study.

## Data Availability

The data underlying this article will be shared upon reasonable request to the corresponding author.
